# Porous silicon-based nanostructured microparticles as degradable supports for solid-phase synthesis and release of oligonucleotides

**DOI:** 10.1186/1556-276X-7-385

**Published:** 2012-07-12

**Authors:** Steven J P McInnes, Nicolas H Voelcker

**Affiliations:** 1 School of Chemical and Physical Sciences, Flinders University, Bedford Park, Adelaide, South Australia, 5042, Australia; 2 Mawson Institute, University of South Australia, Mawson Lakes, Adelaide, South Australia, 5095, Australia

**Keywords:** Porous silicon, Solid-phase DNA synthesis, Microparticles

## Abstract

We describe the preparation of several types of porous silicon (pSi) microparticles as supports for the solid-phase synthesis of oligonucleotides. The first of these supports facilitates oligonucleotide release from the nanostructured support during the oligonucleotide deprotection step, while the second type of support is able to withstand the cleavage and deprotection of the oligonucleotides post synthesis and subsequently dissolve at physiological conditions (pH = 7.4, 37°C), slowly releasing the oligonucleotides. Our approach involves the fabrication of pSi microparticles and their functionalisation via hydrosilylation reactions to generate a dimethoxytrityl-protected alcohol on the pSi surface as an initiation point for the synthesis of short oligonucleotides.

## Background

Single-stranded DNA can be produced in a controlled, stepwise manner [1] using commercial DNA synthesisers on solid supports [1,2]. Once synthesised, the DNA strand is released from the solid support into solution, deprotected and collected (these three steps are usually carried out in aqueous ammonium hydroxide). Side reactions and failure sequences limit the ultimate length of the oligonucleotides to approximately 100 nucleotides. The oligonucleotides produced in this fashion can be used as antisense strands, small interfering RNA and molecular probes. It is also possible to synthesise RNA, locked nucleic acids and peptide nucleic acids using this methodology.

Controlled pore glass (CPG) is the most common solid support for solid-phase DNA synthesis [2], although a number of other supports have also been explored including polystyrene [3,4], carbon [5-7], gold [8,9], as well as glass and silicon [10-14]. However, due to the insolubility of these supports, mechanical separation is required to harvest the newly synthesised DNA. This separation step can result in reduced yields and increased synthesis times [15]. Thus, efforts to improve the efficiency of DNA synthesis have focused on improving the release of DNA. Some research labs have attempted this by focusing on designing new linkers for use on CPG surfaces [16-18], while other labs have attempted to improve the original nucleoside loading of CPG [19] or generate new phosphoramidite and solid-phase chemistry [20]. For example, Azhayev [15] reported on the synthesis of CPG derivatised with long-chain alkyl amino groups, which facilitated faster cleavage of the 3′-terminal phosphodiester group through the action of a secondary hydroxyl group [15].

DNA synthesis has been attempted on porous silicon (pSi) films as well as single crystalline Si(111) and pSi micro- and nano-particles [21-23] for use in electrochemical sensing applications. The combination of DNA and pSi is of interest to biosensor development due to the unique optical and electrical properties of pSi [24-29]. Most recently, Rea et al. [30] have demonstrated the synthesis of a ten-base oligonucleotide directly on two different porous silicon-based Bragg reflectors, one oxidised by a thermal treatment, the other by a chemical process based on exposure to I_2_/pyridine. The synthesis could be monitored by dimethoxytrityl (DMT) release as well as by a red shift in the optical spectra of the Bragg reflector.

However, one of the advantages of pSi, which has so far remained unexplored in the context of solid-phase DNA, is its ability to degrade in aqueous milieu including biological fluids [31,32], which can be exploited for the on-demand release of the synthesised DNA strands from the surface or for the dissolution of the solid support in situ. pSi undergoes oxidative hydrolysis forming silicic acid (Si(OH)_4_), which is non-toxic and comprises nearly 95% of naturally occurring silicon in the environment [33]. It is also possible to exploit the electrochemical fabrication processes in order to control the degradation rates, which in turn could control the release of surface-bound oligonucleotides. pSi can be functionalised on either hydride-terminated or hydroxyl-terminated surfaces with relatively mild reaction conditions and widely available chemicals, allowing further control over the degradation rate [34,35]. Furthermore, pSi can be produced in structures such as microparticles (ranging from tens to hundreds of micrometers). The microparticle size can easily be controlled by the use of sonication [36] and ball milling [37,38].

**Figure 1:**
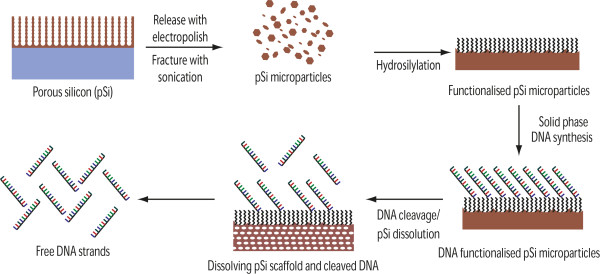
Schematic of the pSi microparticle production and DNA synthesis and release.

Here, we present the synthesis of pSi solid supports with covalently attached linkers, terminally functionalised with a DMT-protected primary alcohol for use as a solid phase for DNA synthesis and, subsequently, the release of the surface-bound oligonucleotides (Figure 1). We use a cleavable linker, based on Azhayev's earlier work, which promotes the release of functional DNA with a 3′ hydroxyl group [15], and a non-cleavable linker based on the hydrosilylation of a long-chain carbon linker, which is subsequently capped with DMT. Depending on the linker chemistry used, the pSi-based solid phase opens opportunities for either the fast deprotection of DNA from a fast degradable support or the release of therapeutic oligonucleotides from a slow degradable delivery system.

## Methods

### Chemicals

Dichloromethane (CH_2_Cl_2_, commercial grade; Merck, North Ryde, Australia) and hexane (commercial grade; Southern Cross, Melbourne, Australia) were distilled over CaH_2_ and stored over molecular sieves away from light. Tetrahydrofuran and toluene (Aldrich, New South Wales, Australia) were distilled over sodium and benzophenone and stored over molecular sieves away from light. Sodium metal (Riedel-de Haen, Seelze, Germany) was used as received after removal of the paraffin oil with hexane. 4-Dimethylaminopyridine (Aldrich), 4,4′-dimethoxytrityl chloride (Fluka, Buchs, Switzerland) and diethylether (analytical grade; Merck) were used as received. Hydrofluoric acid (Merck), MeOH (Merck), acetone (Ajax, Auburn, Australia) and EtOH (Ajax) were used for etching and washing without further purification. N,N-dimethylformamide (DMF; EMD Chemicals, Billerica, MA, USA) was purified, drying over MgSO_4_ followed by distillation at reduced pressure [39]. MilliQ water 18.2 MΩ was obtained from a Labconco, Water Pro PS water purifier (Kansas City, MO, USA). Deuterated chloroform (Cambridge Isotope Laboratories, Inc., Andover, MA, USA) was used without further purification. ω-Undecylenyl alcohol (Aldrich) and 10-undecylenic acid were used as received.

Phosphate buffered saline (PBS) was prepared from sodium chloride (NaCl, 8 g L^−1^; Chemsupply, Port Adelaide, Australia), potassium chloride (KCl, 0.2 g L^−1^; Biolab Scientific, Scoresby, Australia), di-sodium phosphate di-hydrate (Na_2_.HPO_4_^.^2H_2_O, 1.12 g L^−1^; Chemsupply) and potassium dihydrogen orthophosphate (KH_2_PO_4_, 0.24 g L^−1^; Ajax). The pH was adjusted to 7.4 with 1 M NaOH (Ajax) or HCl (Aldrich) in MilliQ water.

### Preparation of pSi microparticles

Microparticles were fabricated from p-type Si wafers (boron-doped, resistivity < 0.001 Ω cm, <1-0-0>) supplied by Virginia Semiconductors (Fredericksburg, VA, USA). The wafer was anodised in an 18-cm^2^ etching cell in 3:1 HF/EtOH solution with a current density of 222 mA cm^−2^ for 4 min, and then electropolished for 30 s at 500 mA cm^−2^. CH_2_Cl_2_ was then added, and the freestanding porous layer was manually fractured into microparticles for collection. Twenty minutes of sonication in dichloromethane was performed (Soniclean 160HT, 70 W, Soniclean Pty. Ltd., Thebarton, Australia) to transform any large sheets of pSi into smaller particles for recovery. The pSi microparticle suspension was filtered and washed with CH_2_Cl_2_ and EtOH. For all subsequent reactions, the pSi microparticle suspension was transferred to a glass reaction vessel containing a fine glass frit (pore size 4.0 to 5.5 μm), and the solvent was removed by aspiration.

pSi microparticles were functionalised via either a cleavable or non-cleavable linker. For full descriptions and schematics of the process, please see Additional file 1. Briefly, fresh pSi microparticles were hydrosilylated with fmoc-11-aminoundecene in mesitylene after deoxygenating with three freeze-pump-thaw cycles. Subsequently, the fmoc-protecting group was removed by shaking with 25% piperidine in DMF before the ring opening of succinic anhydride was performed on the now-exposed surface-bound amines [15]. Following this, any unreacted amines were capped via acylation with acetic anhydride. The carboxy groups introduced by the ring opening of succinic anhydride were then reduced to alcohol groups with the use of LiAlH_4_. Finally, the freshly generated OH groups were capped with DMT. The final surface was named long-chain alkyl amino pSi (LCAA pSi). The non-cleavable linker was produced in a similar fashion via the hydrosilylation reaction of ω-undecylenyl alcohol followed by DMT capping.

### DNA synthesis and characterisation

All DNA synthesis was performed commercially at Geneworks on an Applied Biosystems 394 DNA synthesiser (Applied Biosystems, Life Technologies, Mulgrave, Australia). Coupling reactions were monitored via automated conductivity measurements of the DMT cleavage solution. To calculate the overall yield (OY) (Equation 1) and average stepwise yield (ASWY) (Equation 2), the following formulae [2] were used (where *n* = the number of couplings (or number of bases − 1)).

(1) $$\text{OY}=\left(\text{Lowest conductivity value}/\text{Highest conductivity value}\right)\times 100$$

(2) $$\text{ASWY}={\text{OY}}^{1/n}$$

Two distinct DNA strands were synthesised. For the cleavable linker, a 19mer (3′-CTT GCC GTA GTT CCA CTT G-5′) was synthesised, and for the non-cleavable linker, a 10mer (3′-CTT GCC GTA G-5′) was used.

All DNA synthesis was performed with UltraMild DNA bases purchased from Glen Research (Sterling, VA, USA), and subsequently, DNA deprotection was performed by incubating the pSi microparticles with either potassium carbonate (0.05 M) in MeOH at room temperature for 4 h or ammonium hydroxide at 60°C overnight. Ammonium hydroxide deprotection can be used for both standard phosphoramidites and the UltraMild DNA bases and is the standard protocol for DNA synthesis. The ammonium hydroxide deprotection was used with the cleavable chemistries as the dissolution of the pSi support was desirable and was 100% complete at the conditions used. For the non-cleavable chemistry presented in this manuscript, the mild deprotection with potassium is preferred to conventional ammonium hydroxide as it facilitates the deprotection of the DNA synthesised with UltraMild bases while allowing the DNA to remain bound to the surface (uncleaved) and the pSi to remain intact (undissolved) for subsequent drug release experiments.

### DNA release via UV–vis spectroscopy

UV-visible (UV–vis) spectroscopy was performed on an Agilent 8453 UV–vis spectrophotometer (Agilent Technologies, Santa Clara, CA, USA) using UV-Visible ChemStation Software Rev B.01.01 [21] at a wavelength of 260 nm. Release rates were calculated from the slope of release curves obtained. The actual amount of DNA released was calculated via the use of the Beer-Lambert Law, using the molar extinction coefficient of 194,300 and 90,165 L mol^−1^ cm^−1^ for the 19mer and 10mer, respectively. Molar extinction coefficients were calculated from the nearest-neighbour model at 260 nm using previously reported parameters [40,41]. The amount of DNA released was normalised to the mass of the sample used and then converted to a percentage of the total theoretical payload.

### Ziptip extraction and MALDI-MS analysis

DNA release solutions were purified using 10-μL Ziptip_C18_ pipette tips (Merck Millipore, Billerica, MA, USA). The Ziptips were used according to the manufacturer's protocol. Matrix-assisted laser desorption/ionisation mass spectrometery (MALDI-MS) analysis on the Ziptip-purified DNA was performed on a Waters Micromass M@LDI L/R TOF-MS (Waters Corporation, Milford, MA, USA). The acquisition software was MassLynx 4.1. Analysis was performed in linear mode with the following conditions: polarity, positive ion; source voltage, 15,000 V; pulse voltage, 1,500 V; MCP detector voltage, 1,900 V; laser energy, 89%; matrix suppression, 800 amu; TLF delay, 500.0 ns; and mass range, 1,300 to 15,000 Da.

### Infrared spectroscopy

Infrared (IR) spectra were obtained using a Nicolet Avatar 370MCT (Thermo Electron Corporation, Waltham, MA, USA) equipped with a standard transmission accessory. Spectra were recorded and analysed using OMNIC version 7.0 software in the range of 650 to 4,000 cm^−1^ at a resolution of 4 cm^−1^; the background was taken using a blank KBr disc. IR of pSi microparticles was performed with crushed particles (approximately 2 mg) dispersed in KBr discs. The KBr was supplied by Spectra-Tech (Oak Ridge, TN, USA) and was 99.9 + % FT-IR grade. All IR spectra were collected in transmission mode and subsequently converted to absorbance using OMNIC version 7.0 software. All spectra were normalised using the Si-O peak at approximately 1,100 cm^−1^ and scaling the entire spectra so that the Si-O peak is equal to 1 AU.

### Scanning electron microscopy

Scanning electron microscopy (SEM) was performed on a XL30 field emission SEM (Philips, Amsterdam, Netherlands) at an acceleration voltage of 10 keV. pSi microparticles were dispersed directly onto conductive aluminium stubs for analysis.

## Results and discussion

### Microparticle characterisation

**Figure 2:**
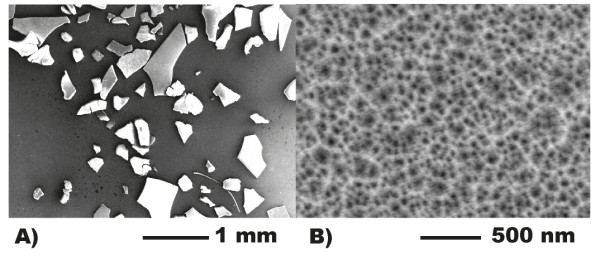
SEM micrographs showing (A) particle size and morphology and (B) pore size.

The porosity of pSi microparticle preparations was calculated by gravimetric analysis [42], and the microparticle thickness and pore sizes were calculated via SEM. The pSi microparticles were found to have porous plate-like structures with a wide range of shapes and sizes (Figure 2A). The porosity was found to be 84.2 ± 2.0%, while the pore size and thicknesses obtained from SEM were 35.5 ± 4.9 nm (Figure 2B) and 30.01 μm, respectively. The particles were sonicated briefly (30 min) to reduce their size, resulting in an average particle size of approximately 87.1 ± 42.3 μm (Figure 2). Despite the dispersion in particle size, further sonication was avoided to ensure that the particles remained large enough to be retained on the glass frit of the columns used on the commercial DNA synthesisers.

**Figure 3:**
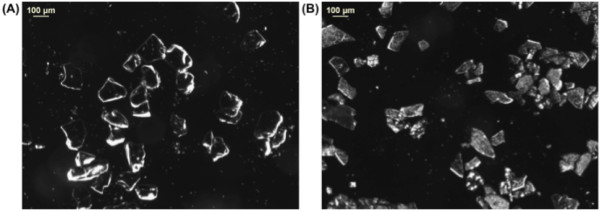
Optical microscope images of (A) CPG and (B) pSi microparticles.

To confirm whether the particles were suitable for DNA synthesis experiments, their size was compared to that of conventional CPG particles. Figure 3 illustrates the size distribution of both the CPG particles (Figure 3A, image count gives 130.5 ± 36.2 μm) and the pSi microparticles (Figure 3B). The pSi possesses a slightly smaller overall particle size. The Applied Biosystems columns used have a pore size of 30 to 40 μm [2] and, hence, can retain both CPG and pSi.

### Cleavable linker chemistry (LCAA) on pSi microparticles

**Figure 4:**
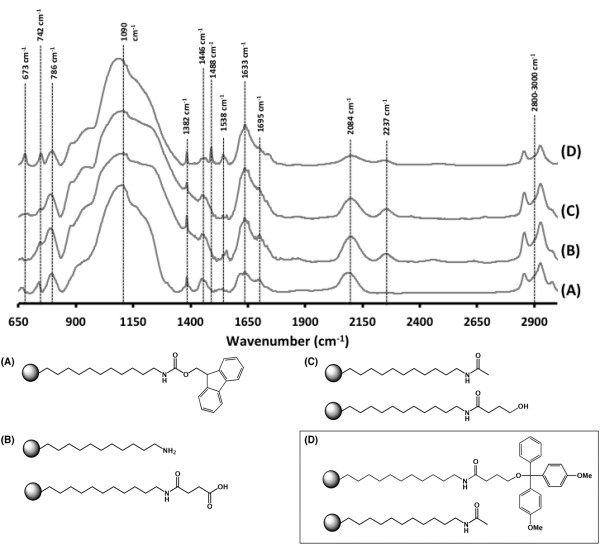
**Comparison of IR absorbance spectra obtained on LCAA-modified pSi surfaces.** (**A**) Hydrosilylated with fmoc-11-aminoundecene, (**B**) after fmoc deprotection and reaction with succinic anhydride, (**C**) capped and reduced with LiAlH_4_ and (**D**) DMT protection of alcohol.

Figure 4 shows a typical transmission IR spectra of each of the synthesis steps, starting with the spectra of pSi hydrosilylated with fmoc-11-aminoundecene (Figure 4A). The notable peaks at 2,084 cm^−1^ for the residual Si-H bonds of Si_3_-SiH groups [43] still remained after the hydrosilylation reaction. However, there were also significant peaks at 1,382 and 1,446 cm^−1^ and a triple peak at 2,800 to 3,000 cm^−1^, indicative of C-H vibrations. The amide bond is indicated by the presence of peaks at 1,633 cm^−1^ (Amide I, C = O stretch) and 1,538 cm^−1^ (Amide II, N-H bending). In the fingerprint region, the peak at 673 cm^−1^ is indicative of C-H out-of-plane deformation vibrations from the benzene, while the two peaks at 742 and 786 cm^−1^ appeared in the fingerprint region and are attributed to C-C skeleton rocking vibration of the long carbon chain. The broad peak centred at 1,090 cm^−1^ is due to asymmetric stretching vibrations of Si-O-Si surface bridging groups caused by oxidation during the hydrosilylation reaction [44]. Similar amounts of oxidation have been reported previously due to the presence of trace amounts of water and oxygen [45-47].

After fmoc deprotection and ring opening of succinic anhydride, the spectra (Figure 4B) still showed the characteristic peaks listed above and new peaks at 1,695 and 2,237 cm^−1^, which were attributed to the new C = O vibrations of the succinic anhydride and possibly by Si-H stretching in O_3_-SiH groups [43]. The IR spectrum of the acetyl-capped and reduced (with LiAlH_4_) surface can be seen in Figure 4C. The only change was the slight decrease of the peak at 1,695 cm^−1^ due to the removal of the carbonyl vibration in the terminal carboxylic acid. The final DMT-capped surface (Figure 4D) showed sharper peaks at 1,382 and 1,446 cm^−1^ as assigned to CH_3_ umbrella deformation and CH_2_ deformation, respectively. Additionally, the reappearance of the peak at 673 cm^−1^ is indicative of C-H out-of-plane deformation vibrations from the benzene. These spectra confirm the presence of the LCAA linker on the surface of pSi.

In order to determine the amount of pSi support required for a standard 40-nmol scale solid-phase DNA synthesis, a pre-weighed amount of the LCAA-functionalised pSi support was reacted with trichloroacetic acid. The thus-generated DMT cation in the supernatant was assayed via UV–vis spectroscopy, and the loading achieved was found to be 13.2 ± 0.3 nmol mg^−1^ of pSi particles, comparable with the loading levels of conventional CPG (15.0 nmol mg^−1^ of resin).

The effect of changing synthesis conditions in terms of coupling, detritylation, oxidation and capping steps during automated DNA synthesis of 19mer (3′-CTTGCCGTAGTTCCACTTG-5′) on LCAA-functionalised pSi particles was investigated to determine optimal DNA synthesis conditions onto LCAA-modified pSi. This investigation was carried out since the OY under standard synthesis parameters was relatively low (41.4%). No significant improvements in OY were seen with extended wait times, doubling the volumes of reagents or both simultaneously. However, doubling of coupling time and volume resulted in a significant improvement, resulting in an OY of 76.6%. The improved yield is possibly attributed to the mass transport limitations experienced on the highly porous material at the standard coupling time and concentration. To confirm this observed increased overall yield, duplicate synthesis was performed with the same parameters, and this synthesis resulted in a final OY of 77.3%, demonstrating a reproducibly high yield. This yield compares well with typical yields seen when short oligonucleotides are synthesised on standard CPG (*ca.* 75% assuming a 99% average stepwise yield) [2].

**Figure 5:**
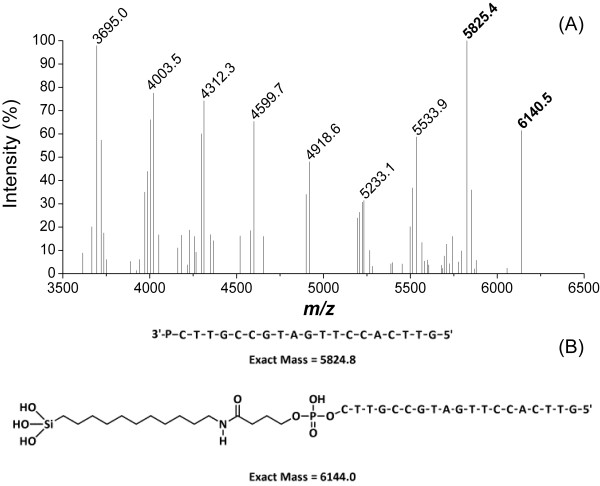
**MALDI-MS spectra of the synthesised DNA.** (**A**) MALDI-MS spectra of DNA synthesised from LCAA-functionalised pSi after deprotection and cleavage. (**B**) Predicted sequence and mass for the 19mer DNA in both fully deprotected state without and with the attached linker.

The synthesised DNA on the pSi support was then treated with 28% ammonium hydroxide at 60°C overnight. These are typical conditions for DNA deprotection and cleavage from CPG. Using UV–vis spectroscopy, we observed that over 99% of the loaded DNA was cleaved from the support. At the same time, the pSi support had completely dissolved. MALDI-MS was then used to confirm the identity of the released DNA. No purification was performed to remove failure sequences. The 19mer without the linker (MH^+^ at *m*/*z* = 5,825.5, expected 5,824.8) and a series of shorter failure sequences can be seen in Figure 5A. A weaker signal for the linker-bound 19mer (see Figure 5B; MH^+^ + Linker at *m*/*z* = 6,140.5, expect 6,144.0) was also observed.

It is important to note that the *m*/*z* values found for both linker-bound 19mer and the free 19mer in Figure 5B are both within ±10 *m*/*z* units of the estimated values. This ±10 *m*/*z* unit window is used commercially by Geneworks to determine if an oligonucleotide is of sufficient quality to sell. The released products shown above indicate that there are definitely two release mechanisms at work, one which produces 3′-phosphate DNA and the other that produces linker-bound DNA. It is possible that the release mechanism producing 3′-phosphate DNA is caused by cleavage of the amide bond by base-catalysed hydrolysis [48], followed by the subsequent elimination of a lactone ring. Meanwhile, the linker-bound DNA is released via the dissolution of the pSi scaffold in strongly alkaline solution [49]. The above approach therefore resulted in a degradable solid support, which was suitable for solid-phase DNA synthesis and which decomposed during the DNA deprotection step.

**Figure 6:**
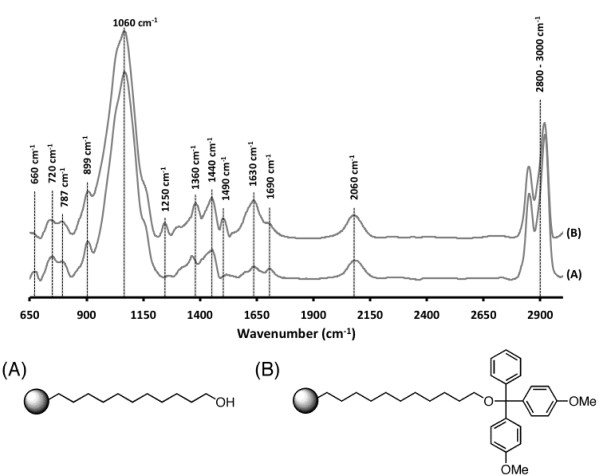
**Comparison of IR absorbance spectra of pSi.** After (**A**) hydrosilylation with ω-undecylenyl alcohol and (**B**) capping with DMT.

### Non-cleavable chemistry

To facilitate the slow release of the full-length DNA from the pSi supports post DNA deprotection, a non-cleavable linker was explored for solid-phase DNA synthesis. This linker was designed to be non-cleavable during the DNA deprotection step and facilitate the release of fully deprotected DNA from the pSi scaffold. The linker was introduced by hydrosilylation with ω-undecylenyl alcohol. The functionalised pSi (Figure 6A) shows significant peaks at 1,360 and 1,440 cm^−1^ and a triple peak at 2,800 to 3,000 cm^−1^ for C-H vibrations. A strong, broad peak centred at 1,060 cm^−1^ due to asymmetric stretching vibrations of Si-O-Si surface bridging groups [44], as well as peaks at 2,060 cm^−1^ due to the residual Si-H bonds remaining after the hydrosilylation reaction, was also observed. After capping with DMT, the pSi surface (Figure 6B) shows new peaks at 1,250 cm^−1^ for C-O-C ether stretching and 1,490 cm^−1^ which corresponds to = C-H and C = C in ring stretching vibrations of benzene rings, demonstrating successful introduction of the protecting group. Alternatively, we were able to introduce the same linker by hydrosilylation of 10-undecylenic acid, followed by reduction with LiAlH_4_ and DMT capping (results not shown).

DMT loading on the pSi microparticles functionalised with the non-cleavable linker was very good, almost four times higher than for standard CPG and the LCAA-functionalised pSi microparticles (56.9 ± 7.4 nmol mg^−1^ compared to 15 nmol mg^−1^).

The synthesis of a 10mer (3′-CTTGCCGTAG-5′) was subsequently performed under optimised solid-phase synthesis conditions. A yield of 66.1% (37.58 nmol of DNA) was obtained, which was only slightly lower than the yield of 10mer for the standard CPG (73.4%).

pSi microparticles functionalised with a 10mer DNA strand and deprotected with 0.05 M potassium carbonate in MeOH retained deprotected DNA on the microparticles, judging from the absence of significant absorbance at 260 nm in the deprotection solution. In contrast, the microparticles functionalised with LCAA chemistry released significant amounts of DNA upon deprotection. The microparticles carrying deprotected DNA were then placed into neutral PBS at 37°C, and DNA release was investigated via measuring the UV–vis absorbance at 260 nm.

**Figure 7:**
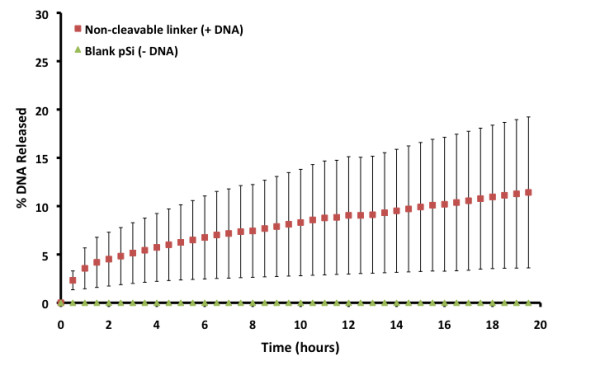
**DNA (10mer) release percentage into PBS from non-cleavable linker-functionalised pSi microparticles (*n*** **≥ 2).**

The release curves for 10mer DNA from pSi microparticles functionalised with the non-cleavable linker were plotted as the percentage of DNA released from the total amount of DNA synthesised on the particles (Figure 7). The surface released 11.4% (4.29 ± 2.94 nmol mg^−1^ DNA of pSi) over a 20-h period (average release rate = 0.23 nmol mg^−1^ h^−1^). These surfaces also showed a small burst release (*ca*. 5%) and a linear release behaviour (*R*^2^ = 0.998) over the following 19-h period. This result suggests that the microparticles are able to sustain the release of DNA for up to 7 days.

**Figure 8:**
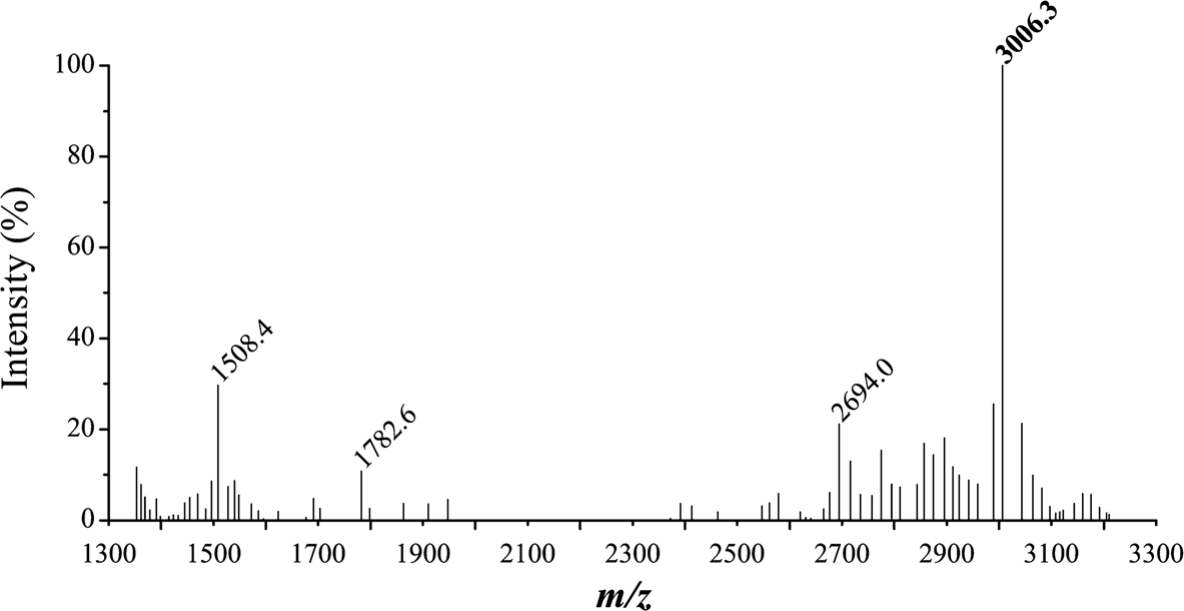
**MALDI-MS spectrum of DNA released from pSi microparticles functionalised with the non-cleavable linker.** After solid-phase synthesis of 10mer DNA and deprotection.

MALDI-MS was utilised to confirm the release of fully deprotected DNA into PBS pH-7.4 solution from the pSi microparticles after 20 h of release (Figure 8). No attempt was made to purify the DNA in order to remove failure sequences. The signal at 3,006.3 *m*/*z* attributed to the released 10mer was the most intense in the mass spectrum and indicates that the DNA is again being cleaved at the 3′phosphate group. Interestingly, with this surface chemistry, the mass spectra showed no linker-bound DNA, indicating the stability of the surface. This increased stability of the surface could be due to the more efficient hydrosilylation of the less sterically hindered ω-undecylenyl alcohol compared to the fmoc-11-aminoundecene used with the cleavable LCAA chemistry. Two failure sequences at 2,694.0 and 1,782.6 *m*/*z* were also observed, corresponding to 3′-CTTGCCGTA-5′ and 3′-CTTGCC-5′ strands, respectively. These strands illustrate that these failure sequences are from the 5′ (growing) end of the DNA. This indicates that the surfaces are stable and do not degrade during the synthesis cycle. The mass spectra also show the doubly charged DNA strands at 1,508.4 *m*/*z*. The results confirm that the DNA released has been fully deprotected and is therefore functional and could be used for therapeutic purposes, for example, in terms of antisense DNA.

## Conclusions

We have described the fabrication of pSi-based supports with both cleavable and non-cleavable chemistries for the solid-phase synthesis of DNA on conventional DNA synthesisers with established reagents and protocols. The cleavable LCAA linker was able to withstand the DNA synthesis protocol with similar OYs to CPG, as well as release 99% of fully deprotected synthesised DNA upon treatment with aqueous ammonium hydroxide solution overnight. The simultaneous dissolution of the solid support is also advantageous as it alleviates the need for a filtration step before purification of the DNA. In contrast, pSi surfaces functionalised with the non-cleavable linker were able to retain the synthesised DNA upon deprotection of the nucleobases, and subsequently released a large amount (4.3 nmol mg^−1^ of pSi) of fully deprotected DNA into PBS at 37°C over 20 h.

The ability to synthesise specific DNA strands on a biodegradable and biocompatible support such as pSi will allow improved control over degradation and drug release profiles. These systems, when combined with a targeting agent, may also be beneficial for the preparation of advanced bio-devices capable of delivering payloads of therapeutic DNA in applications such as gene therapy.

## Competing interests

The authors declare that they have no competing interests.

## Authors’ contributions

SJPM carried out all of the experimental work (except that which was paid for at Geneworks) and drafted the manuscript. NHV conceived of the study and participated in its design and coordination. All authors read and approved the final manuscript.

## Supplementary Material

Additional file 1 **Supporting information.** Full descriptions and schematics of pSi microparticle functionalisation process via either a cleavable or non-cleavable linker [50]. (DOC 394 kb) Click here for file
