# Supplementary material for: Porous silicon-based nanostructured microparticles as degradable supports for solid-phase synthesis and release of oligonucleotides
Source: Nanoscale Res Lett. 2012 Jul 12;7(1):385. doi: 10.1186/1556-276X-7-385 (PMC3552826; doi:10.1186/1556-276X-7-385)
Supplement: Additional file 1 — Supporting information. Full descriptions and schematics of pSi microparticle functionalisation process via either a cleavable or non-cleavable linker [50]. (DOC 394 kb) [file 1556-276X-7-385-S1.doc]

**Porous silicon-based nanostructured microparticles as degradable supports for solid-phase synthesis and release of oligonucleotides**

Steven J. P. McInnes^1,2^ and Nicolas H. Voelcker^2^**[[1]](#footnote-2)***

^1^School of Chemical and Physical Sciences, Flinders University, Bedford Park, SA, Australia, 5042

^2^Mawson Institute, University of South Australia, Mawson Lakes, SA, Australia

5095

Email: nico.voelcker@unisa.edu.au

#### Supporting Information

#### Nuclear magnetic resonance (NMR)

All spectra were obtained on either a Bruker 400 MHz or a Varian 300 MHz NMR as denoted in the text. Solvents used for each samples varied and are denoted in the text prior to the peak assignment listings.

#### Organic synthesis and surface functionalisations

#### Surface build-up of LCAA pSi solid support

##### Synthesis of fmoc-11-aminoundecene

Synthesis of 9-undecenamide (Schematic 1 (1)): A mixture of urea (15 g, 0.25 mol) and undecylenic acid (23.01 g, 0.125 mol) was placed in a round-bottom flask fitted with a condenser. The mixture was heated to 170 - 180 °C for 4 hours. The temperature was then dropped to 110 - 120 °C, saturated Na_2_CO_3_ solution (5 %, 100 - 150 mL) was carefully added through the condenser to stop the reaction. The mixture was then cooled in an ice bath, and the solid product was collected by filtration. The crude material was recrystallised in 95 % EtOH, twice. The final product was dried *in vacuo* to give a colourless solid (11.5 g, 47.9 % yield).

^1^H NMR (300 MHz, *d*-DMSO): *δ* 7.60 (2H, broad, N**H_2_**), 5.86 (1H, m, CH_2_=C**H**), 5.02 (2H, m, C**H_2_**=CH), 2.31 (2H, t, COC**H_2_**), 2.08 (2H, q, CH_2_=CHC**H_2_**), 1.56 (2H, quint, COC**H_2_**C**H_2_**), 1.39-1.30 (12H, m, alkyl-**H**). ^13^C NMR (75 MHz, *d*-DMSO): *δ* 175.0, 138.9, 114.7, 35.7, 33.2, 28.7 (2 overlapping signals), 28.5 (2 overlapping signals), 28.3 and 24.4. ESI-MS: predicted C_11_H_21_NO^+^ (MH^+^) and C_11_H_21_NONa^+^ (MNa^+^) *m/z* = 183.162 and 206.152 respectively. Found *m/z* = 183.169 and 206.151.

Synthesis of 11-undecenylamine (Schematic 1 (2)): LiAlH_4_ (2.7 g, 71.1 mmol) was placed in a 250 mL round-bottom flask containing 50 mL of anhydrous THF, and the mixture was heated at reflux for 30 minutes under N_2_. Heating was stopped and a solution of 9-undecenamide (5.0 g, 27.3 mmol) in 100 mL of anhydrous THF was introduced with stirring. The solution was refluxed under N_2_ for another 24 hours, after which the reaction was cooled, and the excess LiAlH_4_ was subsequently quenched by the dropwise addition of 25 mL of water. The resulting white precipitate was removed by filtration and washed with 3 x 15 mL ethyl acetate. The organic phases were combined and dried over anhydrous Na_2_SO_4_. Evaporation of the solvent followed by vacuum distillation (69 °C at 1.2 mm/Hg) resulted in a colourless oil (1.83 g, 39.4 % yield).

^1^H NMR (300 MHz, CDCl_3_): *δ* 5.74 (1H, m, CH_2_=C**H**), 5.00-4.85 (2H, m, C**H_2_**=CH), 2.61 (2H, t, NH_2_C**H_2_**), 1.98 (2H, q, CH_2_=CHC**H_2_**), 1.36-1.11 (14H, m, alkyl-**H** and 2H, broad s, N**H_2_**). ^13^C NMR (75 MHz, CDCl_3_): *δ* 139.2, 114.1, 42.3, 33.9, 33.8, 29.6, 29.5 (2 overlapping signals), 29.2, 29.0 and 26.9. ESI-MS: predicted C_11_H_23_N^+^ (MH^+^) *m/z* = 169.183. Found *m/z* = 169.190.

Synthesis of (9H-fluoren-9-yl)methyl undec-10-enylcarbamate (fmoc-11-aminoundecene) (Schematic 1(3)): A solution of 11-undecenylamine (0.677 g, 4 mmol) in 30 mL of anhydrous CH_2_Cl_2_ was prepared and cooled in an ice bath. In a separate flask, 9-fluorenylmethoxycarbonyl chloride (1.04 g, 4 mmol) was dissolved in a small amount of anhydrous CH_2_Cl_2_ and was slowly added to the solution of 11-undecenylamine with stirring. After stirring for 2 hours, rotary evaporation of the solvent left an oily white product which was purified by flash chromatography on silica gel with ethyl acetate/hexane (1:4) to yield the product as a white solid (1.28 g, 78.0 % yield).

^1^H NMR (300 MHz, CDCl3): *δ* 7.77 (2H, d, Ar**H**), 7.60 (2H, d, Ar**H**), 7.36 (2H, m, Ar**H**), 5.80 (1H, m, CH_2_=C**H**), 5.02-4.92 (2H, m, C**H_2_**=CH), 4.78 (1H, broad s, N**H**), 4.40 (2H, d, COOC**H_2_**), 4.21 (1H, t, ArC**H**), 3.17 (2H, m, NH-C**H_2_**), 2.03 (2H, q, CH_2_=CHC**H_2_**), 1.37-1.20 (14H, m, alkyl-**H**). ^13^C NMR (75 MHz, CDCl_3_): *δ* 156.4, 144.0 (2 overlapping signals), 141.3 (2 overlapping signals), 139.2,127.6 (2 overlapping signals), 127.5 (2 overlapping signals) 125.0 (2 overlapping signals), 119.9 (2 overlapping signals), 114.2, 66.5, 47.3, 41.1, 33.8, 30.0, 29.5, 29.4, 29.3, 29.1, 28.9 and 26.7. ESI-MS: predicted C_26_H_33_NO_2_^+^ (MH^+^) and C_26_H_33_NO_2_Na^+^ (MNa^+^) *m/z* = 391.251, 414.240. Found *m/z* = 391.259 and 413.241.


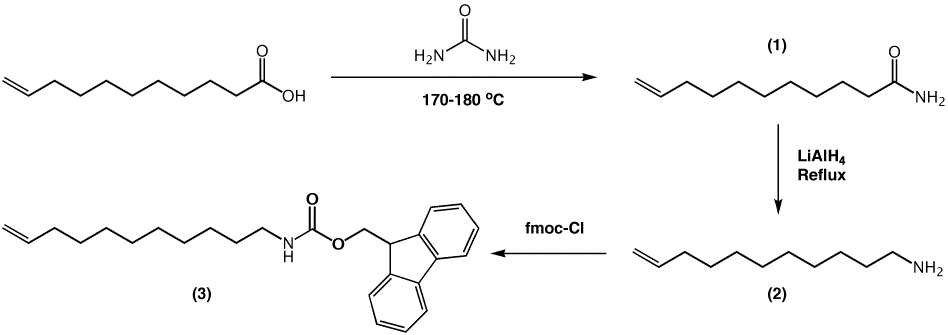


Schematic 1: Overview of reactions performed to generate fmoc-11-aminoundecene.

##### Generation of cleavable long chain alkyl amino pSi (LCAA pSi)

Firstly, fmoc-11-aminoundecene (0.1 M) in mesitylene was mixed with pSi microparticles and subsequently subjected to three freeze-pumped-thaw cycles (consisting of freezing the mixture in liquid N_2_, then pumping for 10 minutes at < 1 mm of Hg followed by thawing under vacuum) to remove all dissolved oxygen. The particles were then reacted for 48 hours at 150 ^o^C. Once cooled, the reaction was washed with mesitylene and THF (Schematic 2, A).

Fmoc deprotection was performed by shaking with 25 % piperidine in DMF for 1 hour at room temperature (RT). After the reaction, the particles were washed with DMF and THF (Schematic 2, B).

The ring opening of succinic anhydride was performed by adding 2.5 mL of pyridine, 2.5 mL of 1-methylimidazole:THF (4:21) and 500 mg (500 micro moles) of succinic anhydride to the particles with shaking overnight at RT. The particles were then washed extensively with THF and dried under vacuum (Schematic 2, C).

Unreacted amines were capped by placing the particles in a mixture of 2.5 mL of 1-methylimidazole:THF (4:21) and 2.5 mL of acetic anhydride:2,6-lutidine:THF (1:1:8) to cap any unreacted amines. The mixture was shaken for 2 hours at RT. The particles were then washed extensively with THF and dried under vacuum (Schematic 2, D).

The COOH groups present on the particles were then reduced via immersion in 10 mL of a solution of LiAlH_4_ (0.2 M in diethylether). The mixture was shaken for 0.5 hours at RT after which the reaction was quenched with 20 mL of 0.4 M sulphuric acid (0.44 mL in 20 mL of H_2_O). The particles were washed extensively with THF and dried under vacuum (Schematic 2, E).

Finally, DMT capping of the OH terminated surface was performed using 10 mL of pyridine containing 500 mg (1.48 mmol) of DMT-Cl and a catalytic amount of DMAP. The mixture was shaken for O/N at RT after which the particles were washed extensively with THF and dried under vacuum (Schematic 2, F).


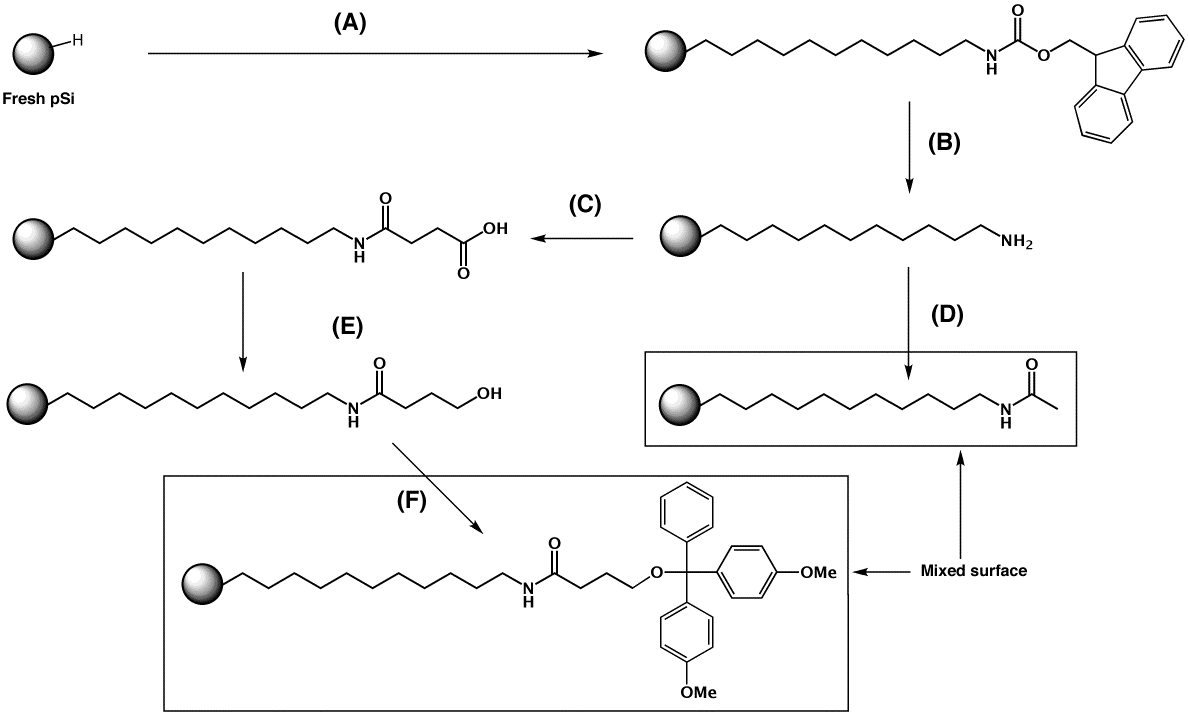


Schematic 2: Overview of reactions generate DMT functionalised cleavable long chain alkyl amino (LCAA) pSi.

#####

##### Conversion of fresh pSi for DNA synthesis with non-cleavable linker

Freshly synthesised pSi microparticles were placed in neat ω-undecylenyl alcohol and freeze-pump-thawed three times to remove all dissolved oxygen. The degassed mixture was then heated to 150 ^o^C and allowed to react overnight. Subsequently, the mixture was cooled and washed with toluene, EtOH and DCM before being completely dried under vacuum. The particles were then immersed in 10 mL of pyridine containing 500 mg (1.48 mmol) of DMT-Cl and a catalytic amount of DMAP. The mixture was shaken overnight at RT after which the particles were washed extensively with THF and dried under vacuum (Schematic 3).


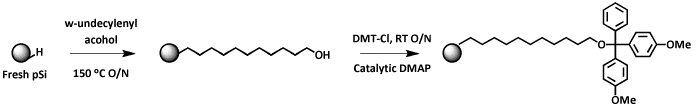


Schematic 3: Overview of reactions performed to generate DMT-functionalised pSi with non-cleavable linkers.

***References***

1. A. M. Ruminski, B. H. King, J. Salonen, J. L. Snyder, M. J. Sailor, *Adv. Funct. Mater.*, **2010**. *20*, 2874–2883.

1. * [↑](#footnote-ref-2)
